# Mapping Free Energy Pathways for ATP Hydrolysis in the *E. coli* ABC Transporter HlyB by the String Method

**DOI:** 10.3390/molecules23102652

**Published:** 2018-10-16

**Authors:** Yan Zhou, Pedro Ojeda-May, Mulpuri Nagaraju, Bryant Kim, Jingzhi Pu

**Affiliations:** Department of Chemistry and Chemical Biology, Indiana University-Purdue University Indianapolis, 402 N. Blackford St., LD326, Indianapolis, IN 46202, USA; zhouyan@gxun.edu.cn (Y.Z.); pedro.ojeda-may@umu.se (P.O.-M.); mulpurinagaraju@gmail.com (M.N.); brykim@iu.edu (B.K.)

**Keywords:** QM/MM, free energy simulations, ABC transporter, ATP hydrolysis, string method, minimum free energy path, proton transfer

## Abstract

HlyB functions as an adenosine triphosphate (ATP)-binding cassette (ABC) transporter that enables bacteria to secrete toxins at the expense of ATP hydrolysis. Our previous work, based on potential energy profiles from combined quantum mechanical and molecular mechanical (QM/MM) calculations, has suggested that the highly conserved H-loop His residue H662 in the nucleotide binding domain (NBD) of *E. coli* HlyB may catalyze the hydrolysis of ATP through proton relay. To further test this hypothesis when entropic contributions are taken into account, we obtained QM/MM minimum free energy paths (MFEPs) for the HlyB reaction, making use of the string method in collective variables. The free energy profiles along the MFEPs confirm the direct participation of H662 in catalysis. The MFEP simulations of HlyB also reveal an intimate coupling between the chemical steps and a local protein conformational change involving the signature-loop residue S607, which may serve a catalytic role similar to an Arg-finger motif in many ATPases and GTPases in stabilizing the phosphoryl-transfer transition state.

## 1. Introduction

As a member of ATP-binding cassette (ABC) transporters [1], Haemolysin B (HlyB) mediates secretion of the 107 kD pore-forming toxin Haemolysin A (HlyA) from gram-negative bacteria in an ATP-dependent manner [2]. Common to all members in the ABC transporter family is a basic architecture consisting of two cytosolic nucleotide binding domains (NBDs) and two transmembrane domains (TMDs). The NBD pair in HlyB is a highly conserved molecular motor that binds and hydrolyzes ATP, energizing the substrate translocation across the TMDs through conformational coupling [3]. In *E. coli* cells, to ensure efficient couplings among these domains, HlyB-NBDs work as an ATPase that catalyzes ATP hydrolysis at a rate constant of *k*_cat_ = 0.2 s^−1^ [3], reducing the half-life of ATP from ~100 days (*k* = 8 × 10^−8^ s^−1^) [4] in aqueous solution to about 3.5 s to meet the kinetic demand of translocating HlyA through bacterial membranes.

**Figure 1:**
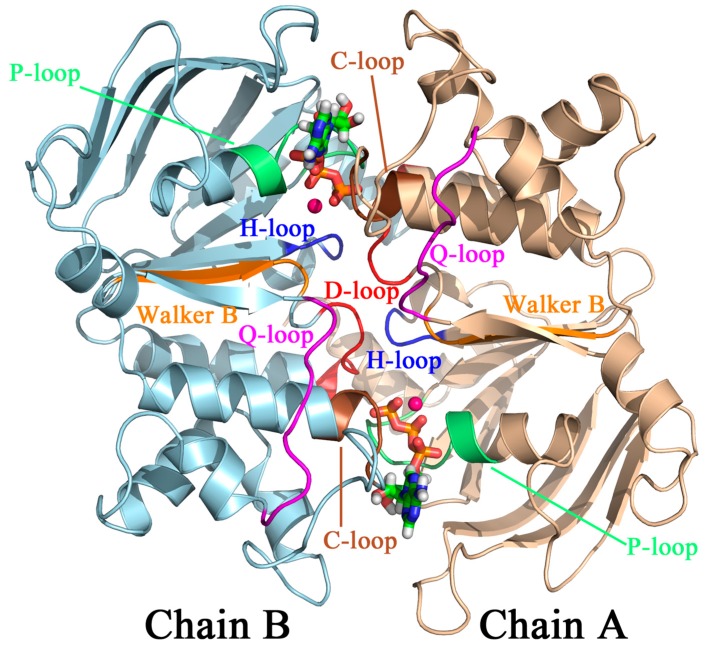
The “head-to-tail” dimeric organization of HlyB-NBDs (cartoon). Two ATP molecules (ball-and-stick) are sandwiched between the P-loop (Walker A) of one NBD and the signature loop (C-loop) of the “*trans*” subunit. Each competent active site also contains the conserved H662 located in the H-loop and the conserved S607 located in the *trans* C-loop.

A molecular-level understanding of how HlyB-NBDs achieve this remarkable catalytic capability has been developed, thanks to a wealth of information accumulated from structural [3,5,6], genetic [7], and biochemical [3,5,6,7] studies. It is known that functioning HylB-NBDs form a dimer (Figure 1). Each NBD monomer adopts an L-shape and can be divided into two subdomains. The RecA-like subdomain (or arm I), consisting of β-sheets flanked by *α*-helices, serves a catalytic core for binding ATP. The catalytic subdomain is then connected to a helical subdomain (also known as the signalling domain or arm II) through flexible loops. The combination of the two subdomains provides a collection of highly conserved sequence motifs deemed essential for ATP hydrolysis in HlyB.

Like many ATPases, HlyB-NBD contains two Walker motifs. The Walker A motif (or P-loop: ^502^GXXXXGKST^510^, where X denotes any amino acid; unless otherwise noted, *E. coli* HlyB sequence numbers will be used throughout this paper), located in the RecA-like subdomain, is mainly responsible for binding the nucleotide phosphate tail, whereas the Walker B motif (^626^ϕϕϕϕD^630^ with ϕ representing any hydrophobic residue) is primarily responsible for Mg^2+^ coordination. Immediately after the Walker B motif is a conserved Glu (E631), which provides a “catalytic carboxylate” that activates the lytic water for a nucleophilic attack of the γ-phosphate group of ATP. In addition, each HlyB-NBD also contributes a signature loop (^606^LSGGQ^610^, also referred to as the C-loop) *trans* to the bound ATP. The C-loop is located in the helical subdomain and unique to the ABC family. Other conserved active site motifs include: the Q-loop, which provides an invariant Gln (^549^LQDN^552^) in one of the linker regions between the RecA-like and helical subdomains; and the D-loop (^634^SALD^637^), which is located near the dimer interface. Overall, the NBD dimer adopts a “head-to-tail” arrangement such that each of the two ATP molecules bound at the dimer interface is sandwiched between the P-loop of one subunit and the C-loop of the opposite subunit (Figure 1).

Despite the great details known about the structural characterization, the precise mechanism by which HlyB hydrolyzes ATP remains unclear. Below, we highlight a few controversial questions that we attempt to answer in the present work.

First, each HlyB-NBD contains an H-loop motif with a conserved His (H662) [3], which is located in its switch II region [8]. Sequence and structural comparisons of ABC transporters and helicases have suggested that the H-loop His may act as a sensor for detecting the γ-phosphate of ATP [7], therefore reminiscent of the conserved Gln in RecA [9]. Mutagenesis studies showed that the mutation of this H-loop His to Ala (H662A) reduces the ATPase activity of HlyB to a background level (<0.1% residual ATPase activity) [3,5]. Similar impairments to catalysis caused by H-loop mutations have also been observed in other ABC transporters, including the histidine permease [10], the maltose transporter [11], and the lipid transporter MsbA [12], although controversial results also exist for the multidrug exporter Pdr5 [13]. The involvement of the H-loop in hydrogen bonding interactions with the P-loop within the same NBD and with the D-loop in the opposite NBD, as revealed in the crystal structures of HlyB, has suggested roles of H662 in ATP binding and in conformational signalling across the NBD dimer interface [3]. Based on a crystal structure of ATP/Mg^2+^ bound dimeric NBDs of HlyB that contains a mutation at the position of H662, Schmitt and co-workers [3] proposed that H662 and E631 form a catalytic dyad, with H662 acting as a “linchpin” that holds a number of key active-site residues at their catalytically competent conformations. Although direct participation of the H-loop H662 in the enzyme mechanism has recently been suggested by us [14] through computation, the functional role of this residue in HlyB catalysis remains elusive.

Another intriguing question is how HlyB (as well as other ABC transporters) manages to function without an apparent “arginine finger” residue that is ubiquitous in many GTP/ATP hydrolyzing enzymes, such as G-proteins [15] (see also Refs. therein) and F_1_-ATPase [16]. For those systems, especially based on knowledge accumulated from GTPase activating proteins (GAPs) [17,18,19,20,21], the notion of “arginine finger” (RF) is often used to rationalize the presence of one or more highly conserved positively charged Arg residues in the active site. Another characteristic of RFs is that they are often provided by a separate function unit (subunit or domain), which can move the finger residues into or out of the nucleotide binding site through large conformational rearrangements. The conformational flexibility associated with the RF movement has been postulated as an important activating mechanism in the related enzymes. One rationale for how they contribute to catalysis is that the positive charges on the mobile Arg residues may preferentially stabilize the buildup of negative charge on the β/γ phosphate groups upon the formation of a dissociative/associative transition state during the phosphoryl transfer step [19,22,23]. In addition to electrostatic stabilization, another possible function of the finger movement has been connected to sensing the change of the chemical state of the γ-phosphate in ATP during hydrolysis. Surprisingly, ABC transporters, including HlyB, catalyze ATP hydrolysis without any apparent active-site RF residues. Although sequence alignment suggests that the Ser residue in the C-loop motif LSGGQ may be placed at the position of an “arginine finger”, [24] it is unclear how a Ser can serve as a substitute for Arg to provide similar electrostatic stabilization to the relevant transition states. Nevertheless, for HlyB-NBD, the ATP/Mg^2+^ bound crystal structure shows that the C-loop Ser (S607) is H-bonded to the γ-phosphate of ATP prior to hydrolysis [3].

**Scheme 1:**
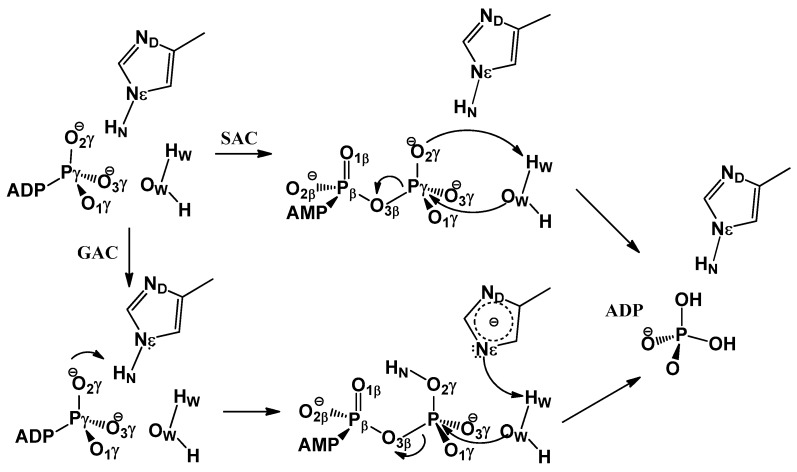
Proposed mechanisms for ATP hydrolysis in HlyB-NBDs. In the substrate-assisted catalysis (SAC) mechanism, a proton is directly transferred from the lytic water to the γ-phosphate oxygen of ATP, whereas in the general acid catalysis (GAC) mechanism, such proton transfer occurs through a relay mechanism, mediated by the H-loop His H662.

In an exploratory study [14], we sought answers to the first question regarding the catalytic role of H662, by using combined quantum mechanical and molecular mechanical (QM/MM) potential energy calculations [25]. In particular, we examined the substrate-assisted catalysis (SAC) mechanism [3], in which the γ-phosphate group of ATP acts as a general base to extract a proton from the lytic water, as well as a new mechanism named by us as the general acid catalysis (GAC) mechanism [14], in which H662 initially serves as a general acid by donating its proton at the N_ε_ position to ATP and subsequently as a general base to activate the lytic water (see Scheme 1 for both mechanisms). A comparison of the corresponding potential energy profiles led us to propose that H662 in HlyB-NBDs may catalyze ATP hydrolysis by facilitating proton transfers via the GAC mechanism. These emerging computational results have suggested a new view of H662 as a “chemical linchpin”, in contrast to the original version of the “linchpin” proposal [3], in which H662 is considered non-reactive and mechanically holds the nearby residues in place through non-bonded interactions.

Although our QM/MM potential energy study benefits from its comparative nature and therefore offers valuable insights into how HlyB works, free energy simulations that statistically sample the reactive system are needed to determine the quantitative features of the enzyme mechanism. Accurate characterization of the free energy profiles for the HlyB reaction would make a more direct comparison between the computed mechanism and the experimental kinetic data possible, potentially helping to explain the observed inverse H/D solvent kinetic isotope effects (KIEs) [3], with which our previous potential energy calculations are not fully compatible [25]. Inclusion of protein dynamics in the simulations is also an important step to characterize the conformational flexibility of S607 and examine its putative finger motion.

We note that after the publication of our original QM/MM potential energy study on HlyB [14], free energy profiles for ATP hydrolysis in MalK_2_, which is the NBD in another ABC transporter, have been reported by Huang and Liao [26] and by Hsu et al. [27]; although both these pioneering works seem to support a dissociative-like mechanism, with proton transfer to the H-loop His also being observed, they yield quantitatively very different free energy profiles. These discrepancies may be linked to the system dependency of the mechanism, as well as the sensitivity of the computational methods used, and indicate the challenging and inconclusive nature of QM/MM free energy simulation for ABC transporters, which is still in its infancy.

In this work, we report our own QM/MM free energy simulations of the HlyB reaction. Because both experiments [28,29] and calculations [14] suggest that E631 is unlikely to be the catalytic base in HlyB, here, we only focus on the SAC and GAC mechanisms, which use ATP and the H-loop His, respectively, for proton abstraction from the lytic water. The minimum free energy pathways (MFEP) of ATP hydrolysis for both mechanisms are obtained by using the string method [30,31]. Although especially suitable for studying free energy profiles of complex conformational transitions using coarse-grained [32,33] or all-atom molecular mechanical potentials [31,34,35], the string method has also gained popularity in recent studies of enzyme reactions when used with QM [36] or combined QM/MM potentials [37,38,39,40,41,42]. In addition to confirming the catalytic role of the H-loop H662 through QM/MM free energy simulations, the impact of the C-loop Ser (S607) on catalysis is also investigated here through in silico mutation simulations.

The rest of the paper is organized as follows. In Section 2, we provide the theoretical background and computational details of the QM/MM free energy pathway simulations. The results and discussion are presented in Section 3. Unresolved issues in this study are further discussed in Section 4. Concluding remarks are given in Section 5.

## 2. Methods

### 2.1. Theory: MFEP by the String Method in Collective Variables

In the string method, a path on the free energy surface is expressed as a one-dimensional curve in a set of *N* collective variables (CVs) **θ**[**x**] = (*θ*_1_[**x**], *θ*_2_[**x**], …, *θ_N_*[**x**]), where **x** denotes the Cartesian coordinates of atoms. The path is then given by describing how the values of CVs, denoted **z**, with **z**[*α*] = (*θ*_1_ = *z*_1_[*α*], *θ*_2_ = *z*_2_[*α*], …, *θ_N_* = *z_N_*[*α*]), evolve along the string as a function of a scalar parameter *α*. The so-called minimum free energy path (MFEP) condition is achieved when the forces of the free energy *G*(**z**) on the collective variables, $f=-\nabla_{z}G(z)$, are found tangent to the path everywhere. In practice, the string path can be represented by *R* discrete copies (referred to as “images”) of the system and parametrized by a reduced arc-length *α* ∊ [0, 1], based on the Euclidean metric calculated in the CV space, such that the string evolves from reactant (*α* = 0) to product (*α* = 1). To prevent the images from falling into the nearby local minima, the so-called reparametrization step is carried out following each path-optimization step to re-distribute the images along the path so that all adjacent images are always separated by a uniform distance of Δ*α* = 1/(*R* − 1). The cycle of optimization and reparametrization repeats iteratively until the string path converges.

As shown by Maragliano et al. [31], the free energy gradients $\nabla_{z}G(z)$ (negative mean forces) needed for string path optimization can be estimated from fluctuations of the CVs using a set of restrained molecular dynamics (MD) simulations of the images, in which each CV is harmonically anchored at a corresponding reference value:

(1) $$\frac{dG(z)}{dz_{i}}≅\frac{k}{T}\int_{0}^{T}\left\{z_{i}-\theta_{i}[x(t)]\right\}dt$$

where *k* denotes the force constant of the harmonic restraint, *T* is the simulation time for estimating the statistical average of the free energy force, and *z_i_* is the reference value at which the *i*th collective variable *θ_i_* is restrained.

When curvilinear internal coordinates are used in the collective variables to represent the string path, the stationary condition of MFEP is generally expressed as:

[**M**(**z**)**f**(**z**)]^┴^ = 0 (2)

where ^┴^ denotes the vector components orthogonal to the path, obtained by applying a projection operator. Note that the metric tensor **M**(**z**) in Equation (2) (or equivalently the diffusion tensor [33] in a derivation based on Brownian dynamics of CVs) is needed to take the curvilinear nature of the path into account. In the string implementation using restrained MD simulations, the elements of the metric tensor can be estimated using a time average [31]:

(3) $$M_{ij}≅\frac{1}{T}\sum_{k=1}^{n}\int_{0}^{T}\frac{\partial \theta_{i}[x(t)]}{\partial x_{k}}\frac{\partial \theta_{j}[x(t)]}{\partial x_{k}}dt$$

where *x_k_* denotes the *k*th-Cartesian coordinate (out of *n* Cartesians in total) involved in the computation of each collective variable, and *T* represents the length of the MD trajectory for the time average. Finally, the free energy profile *G* as a function of **z**[*α*] is obtained by integrating the free energy gradient (also making use of chain rule) along the MFEP (see Maragliano et al. [31] for details):

(4) $$G(z[\alpha ])=G(z[0])+\int_{0}^{\alpha }\sum_{i=1}^{N}\frac{dz_{i}(\alpha^{\prime })}{d\alpha^{\prime }}\frac{dG(z[\alpha^{\prime }])}{dz_{i}}d\alpha^{\prime }$$

Note that although an alternative definition of the free energy profiles exists, which relies on more rigorous integration over the scalar variable *α* using techniques such as Voronoi tessellation, [43], we focus on the free energy defined by Equation (4) in the present study, considering that the difference between these free energy definitions is expected to be small for the relatively narrow transition/reaction tubes [34,43] often seen in chemical reactions [38].

### 2.2. Computational Details

#### 2.2.1. Structural Model, Potential Energy Surface, and General MD Setup

**Figure 2:**
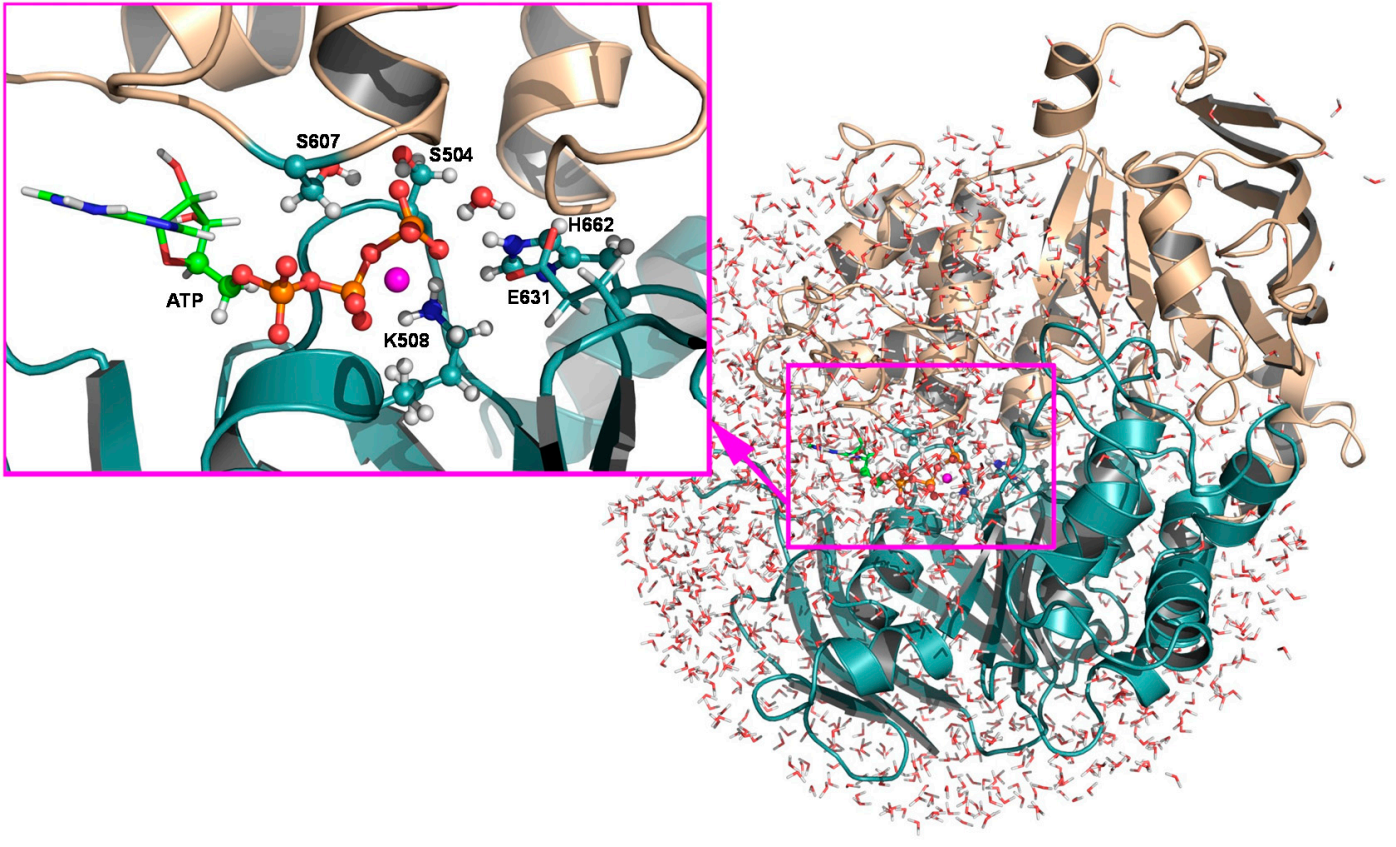
Stochastic boundary setup of the simulation system, where the active site is solvated by a water sphere of 30.4 Å, centered at the γ-phosphorous atom of ATP. Close-up of the QM region, which consists of three phosphate groups of ATP; side chains of S504, K508, and H662 in chain B of HlyB-NBDs; the side chain of S607 in chain A; the lytic water; and five GHO boundary atoms. The components shown in the licorice representation are originally located in the 1XEF structure, except side chains of H662 and E631, and the lytic water, which were modeled from the 2FGK structure. This figure was generated by PyMOL.

We constructed the HlyB-NBD dimer based primarily on its ATP-bound H662A mutant structure crystalized in the presence of Mg^2+^ (PDB: 1XEF) [3], except that the side chains of H662 and E631 were modelled using the structure of the ATP-bound E631Q mutant (PDB: 2FGK) [6]. As shown in our previous work [14], the combination of the two structures is important for obtaining the catalytically active form of the motor domain. The dimer was then solvated in a 30.4 Å sphere of modified TIP3P waters [44] (Figure 2), with two chloride ions added to neutralize the overall charge of the system. An MOPAC-based [45] combined QM/MM [46] method implemented in the CHARMM [47] program was employed to define the potential energy surface (PES), on which free energy path sampling was carried out. The simulation system was centered at the γ-phosphorous atom (P_γ_) of ATP in chain B of the modelled structure and divided into two subsystems. A subsystem of 61 atoms, consisting of the triphosphate group, as well as the C4′, C5′, H5′, H5′′, and O5′ atoms of ATP; the side chains of S504, K508, and H662 in chain B; the side chain of S607 in chain A; and the lytic water (W_L_), is treated quantum mechanically at the semiempirical Austin Model 1 (AM1) level [48]. The rest of the system, including the protein environment and the bulk solvent, is described molecular mechanically by the CHARMM22 force field [44] with the CMAP corrections [49]. Finally, the QM and MM regions are joined through five boundary atoms treated quantum mechanically by the generalized hybrid orbital (GHO) approach [50] (Figure 2). As shown in our previous study, the AM1/CHARMM22 combination generates reasonable energy profiles for the HlyB reaction [14]. Therefore, the same level of theory is chosen here for validation of the mechanism when entropic contributions are included. In addition, the use of semiempirical Hamiltonian offers the computational efficiency needed for conducting adequate statistical sampling in QM/MM free energy simulations.

A group-based scheme was used to determine the non-bonded pairs with a cutoff distance of 17 Å, based on which the non-bonded interactions were evaluated by applying a force switch function in the region of 15–16 Å. The dynamics of the locally solvated system were described using a stochastic boundary condition [51] treatment. More specifically, a sphere of 24 Å from the center of the solvated active site was selected as the reaction region, in which atoms were simulated by Newtonian dynamics. Atoms located in the buffer region, defined as the layer between 24 and 30.4 Å from the center, were treated by Langevin dynamics. Ref. [52] Atoms beyond the buffer region were kept fixed. Bonds involving hydrogens in the MM region were constrained using the SHAKE algorithm [53] during MD simulations.

#### 2.2.2. String Simulations

As our preceding paper [14] shows, at least two chemical processes, namely, proton transfer and phosphoryl transfer, need to be monitored to describe the overall ATP hydrolysis reaction in HlyB. The bond rearrangements in these processes are therefore included in the definition of the string paths. Specifically, for the GAC mechanism, a set of six collective variables, including the bond distances of H_N_-N_ε,H662_, H_N_-O_2γ_, H_W_-O_W_, H_W_-N_ε,H662_, P_γ_-O_3β_, and P_γ_-O_W_, was used in the MFEP simulations (see Scheme 1 for atom labels). By contrast, only four collective variables, i.e., the distances of P_γ_-O_3β_, P_γ_-O_W_, H_W_-O_W_, and H_W_-O_2γ_, were used for the SAC mechanism. For simulations of both mechanisms, a total of *R* = 25 images were placed along each string to describe the MFEPs. The initial string, denoted **z**_0_, and the corresponding initial structures for each image, were taken from the minimum energy paths (MEPs) obtained previously on the two-dimensional PESs at the consistent AM1/CHARMM22 level [14]. Each initial image was first heated up gradually to 298 K during 60 ps, followed by an equilibration simulation of 100 ps at 298 K, before proceeding to MFEP optimization.

To converge the MFEPs, we performed in each case 100 cycles of string path optimization. During each path-optimization cycle, we carried out 20 ps constant temperature MD simulations at 298 K (temperature of the reaction region is maintained through a Berendsen thermostat [54]), with an integration step of 1 fs to sample the image systems whose CVs are harmonically anchored at their corresponding path values. The last 15 ps of the trajectories from each restrained MD simulations were used to compute the average free energy gradients on the CVs according to Equation (1). The same type of time average was also used to obtain elements of the metric tensor matrix **M** based on Equation (3). Necessary modifications were made in a local version of the CHARMM [47] program to obtain the time evolution of each CV’s deviation from their reference values **[θ**(*t*)-**z**] and that of their partial derivatives ∂*θ*/∂*x_k_* with respect to related Cartesian coordinates *x_k_*. Altogether, a total of 50 ns (20 ps/image × 25 images/cycle × 100 cycles) QM/MM MD simulations were performed to obtain an optimized string path.

During each cycle of path optimization, once the mean forces on the collective variables were obtained, the string was evolved following steepest-descent dynamics [31], which updates the values of CVs at each image with a scalar time step of Δ*t* = 0.001. The tensor matrix that accounts for the curvilinear nature of the string path and the projection operator used for Equation (2) were constructed following the original reference of Maragliano et al. [31]. After each step of the steepest-descent update, the string path was first smoothed [31] among adjacent images (with a smoothing parameter of *s* = 0.01) and then reparametrized by using a linear interpolation scheme [31], which re-distributes the 25 images over path segments of equal Euclidean arc-length. The final free energy profile along the MFEP was determined by numerical integration of Equation (4) using a trapezoidal rule along a grid of 75 points over intervals of [0, *α*] (with *α* ∈ [0,1]), where the free energy mean forces and derivatives for coordinate transformation on additional points between the adjacent images were obtained by cubic splines [55].

Convergence of the string MFEPs was monitored by the root-mean-square-deviation (RMSD) between the CV values obtained in a given path-optimization cycle and those obtained in the last cycle, averaged over all images along the path. We found that the MFEPs computed here for the HlyB system converge well after 90 cycles of path optimization (with RMSDs < 0.01 Å) (see Section 3.4 for details). Therefore, further optimization cycles only modify the resulting free energy profiles due to statistical fluctuations of the free energy mean forces. Based on this observation, we estimated the statistical errors in free energy using the standard deviations of the free energy profiles obtained during the last 10 cycles (i.e., cycles 90 to 100) of string path optimization.

## 3. Results and Discussion

**Figure 3:**
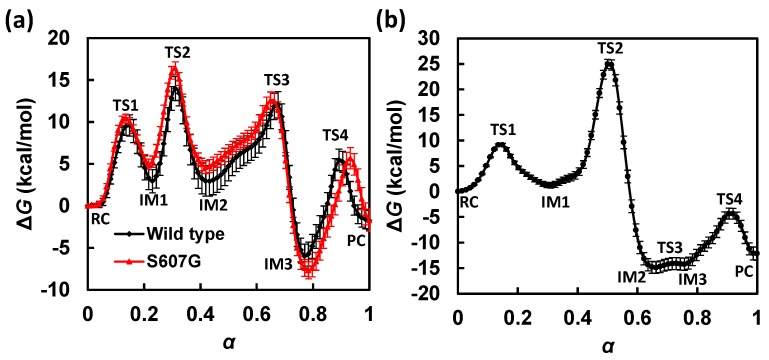
Comparison of free energy profiles along the minimum free energy paths (MFEPs) for (**a**) the GAC and (**b**) the SAC mechanism. The GAC free energy profile for the S607G mutant is also shown in (**a**) as red (grayscale = gray). The statistical fluctuations of free energy are estimated based on the standard deviations of free energy profiles obtained during the last 10 cycles (i.e., cycles 90 to 100) of string path optimization.

**Figure 4:**
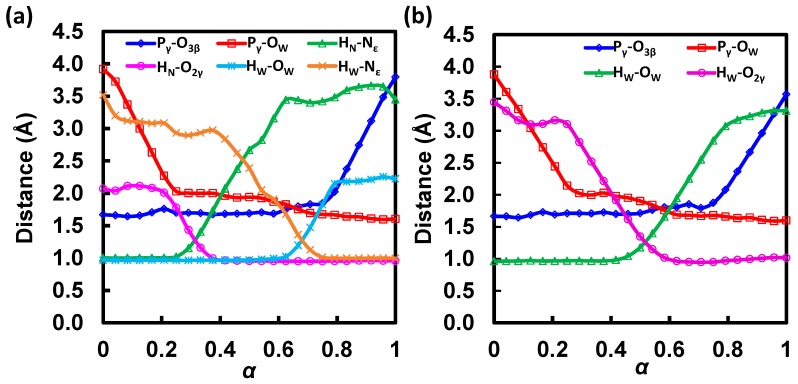
Progressions of collect variables along the MFEPs for (**a**) the GAC and (**b**) the SAC mechanisms in ATP hydrolysis catalyzed by HlyB-NBDs obtained at the AM1/CHARMM22 level.

**Figure 5:**
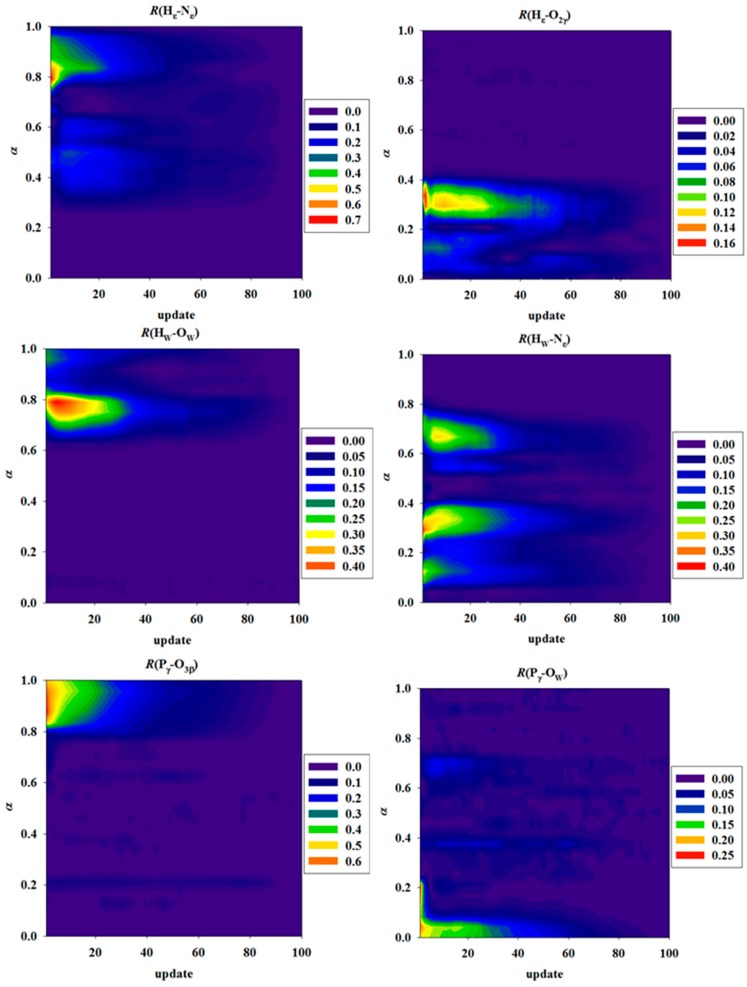
Convergence of the string MFEPs for the GAC mechanism, monitored by the deviations of collective variables (CVs) from their values obtained in the last cycle (i.e., the 100th cycle) of string path optimization. In each panel, the deviations (in Å) for the corresponding bond-distance-based CV in the GAC mechanism are represented by color maps (see color bars for scale) as a function of the number of path optimization cycle (*x*-axis) and the scalar parameter *α* (*y*-axis) which measures the position of each image along the string path.

**Figure 6:**
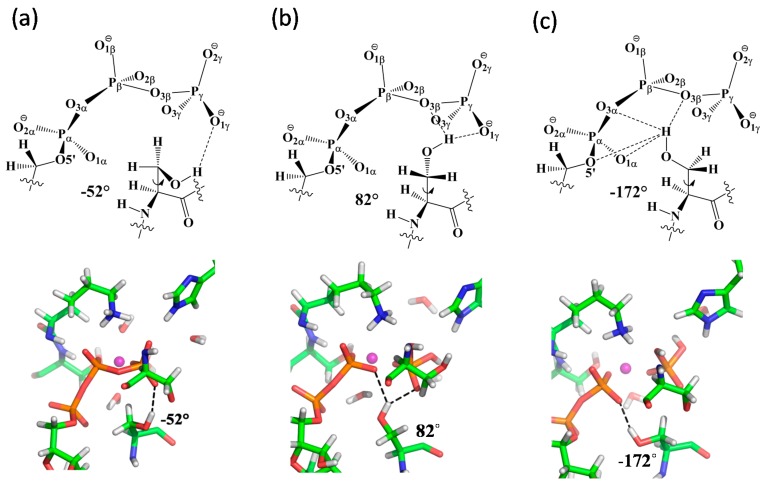
Patterns of H-bonds and representative geometries for the three conformations of the C-loop S607 observed during ATP hydrolysis: in the (**a**) reactant, (**b**) associative phosphoryl transfer transition state (TS3), and (**c**) product regions. The “finger” motion of S607 associated with the three conformations can be characterized by a transition of the dihedral angle *d*(C-C_α_-C_β_-O_γ_) in the residue from −52°, through +82°, to −172°.

The free energy profiles along the MFEPs obtained for the GAC and SAC mechanisms are shown in Figure 3. The transition states located on the MFEPs are further verified by computing the committor probabilities based on short trajectories launched from the corresponding transition state ensembles (see Supplementary Materials SM.1 for *Committor Analysis*). Progressions of the CVs along the MFEPs are plotted in Figure 4. The numerical values of free energy for the stationary states located on the MFEPs, as well as the corresponding CV values at each state, are given in Table 1 and Table 2, for the GAC and SAC mechanisms, respectively. Convergence of the CVs in MFEP optimization is shown in Figure 5 for the GAC mechanism. The H-bond patterns and representative geometries for three distinct conformational populations of the C-loop S607 found during ATP hydrolysis are displayed in Figure 6. Next, we describe the major features of the free energy profiles obtained for the two reaction mechanisms.

### 3.1. Free Energy Profile for GAC

In the MFEP determined for the GAC mechanism (Figure 3a, black; Table 1), the first transition state (TS1; *α* = 0.15) is associated with the lytic water’s approaching ATP and H662. An encounter complex intermediate (IM1) is formed between ATP and the lytic water at *α* = 0.23. Compared with those in the reactant complex (RC) at *α* = 0.00, both distances from the lytic water to the γ-phosphate and to H662 decrease in the encounter complex; this can be seen from the shortened P_γ_-O_W_ (from 3.92 Å in RC to 2.15 Å in IM1) and H_W_-N_ε,H662_ (from 3.52 Å in RC to 3.01 Å in IM1) distances in Figure 4a, which suggest formations of stronger hydrogen bonds between the lytic water and ATP, as well as H662 in IM1.

Following the formation of IM1, the O_2γ_ atom in ATP serves as a general base and abstracts the H_N_ proton from H662. During this proton transfer, the orientation of the lytic water is anchored by a strong hydrogen bond to E631 and a weak hydrogen bond to the N_ε_ atom of H662. After H_N_ is transferred, the complex formed between the protonated ATP and the lytic water becomes even more compact. While still hydrogen bonded to E631, the lytic water forms a stronger hydrogen bond with H662 due to the loss of the H_N_ proton at the N_ε_ position. Based on the free energy profile (Figure 3a, black; Table 1), this proton transfer step using H662 as a general acid is identified as the rate-limiting step in the GAC mechanism; the corresponding transition state (TS2; *α* = 0.31) is associated with a free energy barrier of Δ*G*^‡^ = 14.0 kcal/mol.

Upon the completion of proton abstraction from H662 to ATP (IM2; *α* = 0.43), the lytic water approaches H662 and loses contact with E631. During the transition from IM2 to IM3, the O_2γ_-H_N_ group rotates away from H662 and forms hydrogen bonds with O_3β_ and O_1γ_. In the same transition, the H_W_ proton of the lytic water, which is originally hydrogen bonded to the N_ε_ atom of H662 through non-covalent interaction, starts to migrate to N_ε_. During this second proton transfer step, the P_γ_-O_W_ bond is also being formed. The transition state for this concerted proton transfer/P_γ_-O_W_ bond formation step, TS3 (*α* = 0.68), involves a pentacovalent P_γ_ configuration. This can be seen from a change of the dihedral angle *d*(O_1γ_-P_γ_-O_2γ_-O_3γ_) from ~140° in IM2 to ~180° in IM3, which indicates the formation of a planar γ-phosphoryl group. In the intermediate IM3 (*α* = 0.77), the H_W_-N_ε,H662_ bond has been well-formed, as seen from its bond distance of 1.01 Å, and the P_γ_-O_3β_ bond is broken, with its bond length elongated to 1.93 Å. It is shown on the free energy profile that IM3 is the most stable intermediate (with a free energy of −6.0 kcal/mol with respect to RC), perhaps due to the strong hydrogen bonds between O_W_ or O_2γ_ and the proton on N_ε_, as well as the formation of the adenosine diphosphate/inorganic phosphate complex (ADP·Pi).

The final step is the departure of Pi from ADP, with O_3β_ maintaining its coordination to the Mg^2+^ ion. In TS4 and the product complex (PC; *α* = 1.0), O_2γ_-H_N_ forms a strong hydrogen bond with O_3β_. In PC, Pi is also stabilized by the Walker B residue D630, which forms a hydrogen bond with the remaining proton on O_W_. We note that prior to PC formation, D630 has been H-bonded to the side chain of S509 (a P-loop residue), as well as two non-lytic solvent waters, and is therefore well-shielded from the O_W_ atom. In PC, the two non-lytic waters are displaced by a local structural change, which also exposes the carboxylate group of D630 to accept an H-bond from O_W_-H_W_ in Pi. PC has a free energy of −1.7 kcal/mol, suggesting that ATP hydrolysis in the studied NBD active site is not a major chemical free energy releasing step; little free energy loss after hydrolysis may be beneficial for efficient chemomechanical coupling in converting chemical free energy to mechanical work through coulomb repulsion subsequently developed during ADP/Pi separation [56].

### 3.2. Free Energy Profile for SAC

The free energy profile along the MFEP obtained for the SAC mechanism is shown in Figure 3b (see also Table 2 for free energy and CV values at the stationary states). The first step is the lytic water approaching, associated with a free energy barrier of 9.1 kcal/mol (TS1; *α* = 0.14). An ATP and lytic water encounter complex (IM1; *α* = 0.31) is formed after the first step, in which the P_γ_-O_W_ bond is shortened from 3.88 Å in RC (*α* = 0.00) to 2.01 Å. The second step corresponds to proton transfer from the lytic water to the O_2γ_ atom of ATP and a concerted formation of the P_γ_-O_W_ bond. This step is the rate-limiting step for the SAC mechanism and associated with a free energy barrier of Δ*G*^‡^ = 25.0 kcal/mol (TS2; *α* = 0.50). In TS2, the proton is transferred halfway between its acceptor and donor, and the transition state is in nature associative as the P_γ_-O_W_ bond is partially formed with a bond length of 1.90 Å, while the P_γ_-O_3β_ bond distance is well-kept at 1.71 Å (Figure 4b; Table 2). Following the proton transfer step is the formation of two pentacovalent intermediates (IM2 at *α* = 0.66 and IM3 at *α* = 0.76), which are similar in free energy. The two intermediates are separated by a small free energy barrier (<1 kcal/mol; TS3 at *α* = 0.73), which can be ascribed to a local hydrogen bonding rearrangement from IM2 to IM3. The O_2γ_-H_W_ group, originally oriented toward the nucleophile in IM2, rotates by ~180° about the axis along the P_γ_-O_2γ_ bond such that it points to the leaving group in IM3, which creates an intermolecular H-bond with the β-phosphate of ATP. The last step is the cleavage of the P_γ_-O_3β_ bond, which leads to the formation of the final ATP hydrolysis product complex (PC; *α* = 1.0). Based on our simulations, the cleavage step is associated with a non-substantial free energy barrier (TS4; *α* = 0.91), which is not rate determining in the SAC mechanism.

Under the SAC mechanism, the overall free energy of ATP hydrolysis in the HlyB-NBD active site is −12.2 kcal/mol; this exergonicity is much closer to the unperturbed chemical equilibrium for ATP hydrolysis in aqueous solution which significantly favors the hydrolysis products.

### 3.3. GAC Is Preferred over SAC for Catalysis

The free energy profiles for the GAC and SAC mechanisms are juxtaposed in Figure 3 for comparison. Despite the fact that the reaction is computed in the enzyme environment, the rate-determining step for the SAC mechanism gives a substantial free energy barrier of 25.0 kcal/mol (SAC:TS2), which is nearly as high as that for ATP hydrolysis in aqueous solution (based on a rate constant of *k* = 8 × 10^−8^ s^−1^ [4], the free energy barrier for the non-catalyzed solution-phase reaction is estimated to be 27.4 kcal/mol). This result, together with our earlier two-dimensional QM/MM potential energy study [14], suggests that the SAC mechanism is unlikely to be the operative ATP-hydrolysis mechanism in HlyB. By contrast, for the GAC mechanism, which allows H662 to assist proton relay, the highest free energy barrier is only 14.0 kcal/mol (GAC:TS2), in good agreement with a free energy barrier of 18.6 kcal/mol estimated from the experimental *k*_cat_ of 0.2 s^−1^ for HlyB [3]. Thus, the new free energy results in this work provide further evidence in support of the “chemical linchpin” proposal [14], as recruiting H662 for explicit participation in the HlyB-NBD ATPase mechanism is shown to reduce the rate-limiting free energy barrier by ~10 kcal/mol compared with the direct proton transfer pathway.

Comparing the free energy results with the potential energy results obtained previously, we found that the incorporation of entropic contributions (from dynamics of ligand, protein, and solvent) tends to lower the overall barriers. Specifically, the barrier associated with TS2 in the SAC mechanism is reduced from 32.1 (potential energy; Zhou et al. [14]) to 25.0 kcal/mol (free energy; this work). Although we see some quantitative difference in these barriers, the qualitative feature of the SAC mechanism remains unchanged: TS2 is still identified as the rate-determining transition state over the reaction process, in line with the prediction from our earlier potential energy calculations [14]. For the GAC mechanism, the free energy and potential energy calculations generate a similar barrier height of 14.0 and 15.0 kcal/mol [14] at TS2, respectively, whereas the barrier at TS3 is reduced from 22.1 (potential energy; Zhou et al. [14]) to 12.0 kcal/mol (free energy; this work; see also Table 1). Consequently, the rate-limiting step is moved from TS3 to TS2 in the GAC mechanism when free energy is considered.

It is worth noting that the barriers associated with formations of the associative phosphoryl transfer intermediates in both mechanisms, i.e., TS2 in SAC and TS3 in GAC, are greatly reduced by ~7–10 kcal/mol when entropic effects are included. Because substantial geometric rearrangements in the ligand are involved in the formations of these associative phosphoryl transfer transition states, the greater reduction of SAC:TS2 and GAC:TS3 barriers may reflect additional transition-state stabilization due to dynamical motion of the protein environment, especially that of the conformationally flexible signature-loop residues in the active site (see Section 3.5 below). By contrast, for TS2 in GAC, which only involves proton relocation between the somewhat rigid H662 and ATP moieties, dynamics do not seem to modify the corresponding barrier significantly. Despite a different assignment of the rate-determining step in the GAC mechanism, a comparison of the free energy profiles for the two mechanisms confirms our previous conclusion based on QM/MM potential energy calculations, which favors the GAC over the SAC mechanism for HlyB-catalyzed ATP hydrolysis. Interestingly, these new free energy results seem to reconcile computation with the experimentally observed inverse H/D solvent kinetic isotope effects (KIEs) in HlyB [3], as the rate-determining free energy barrier obtained for the preferred mechanism, i.e., TS2 in GAC, does not involve proton transfer from the lytic water (see Section 4.3 for details).

The rate-limiting step in the SAC mechanism is the step of transferring H_W_ to O_2γ_ (coupled with the P_γ_-O_W_ bond formation), through a four-center transition state TS2. The involvement of H662 converts this single proton transfer step into two consecutive steps that relay two protons, thereby avoiding the geometrically strained four-center reaction [57] and reducing the free energy of activation required for the protonation of O_2γ_ by ~10 kcal/mol [ΔΔ*G*(TS2^SAC^-TS2^GAC^)]. The present results also suggest that the HlyB enzyme may utilize protein dynamics to stabilize the transition state of P_γ_-O_W_ bond formation (TS3 in GAC), so that the enthalpic barrier associated with phosphoryl transfer is greatly lowered, effectively shifting the dynamical bottleneck to the protonation of O_2γ_.

### 3.4. Convergence of String MFEPs

To check convergence of the string paths during MFEP optimization, we monitored the differences between the CV values obtained for each image in a specific path-optimization cycle and those obtained in the last cycle. The deviations in terms of the six bond distances, namely, **θ**^GAC^ = (H_N_-N_ε,H662_, H_N_-O_2γ_, H_W_-O_W_, H_W_-N_ε,H662_, P_γ_-O_3β_, P_γ_-O_W_), which define the CVs used in determining the MFEPs for the GAC mechanism, are shown in Figure 5 using a color-map representation. Although the convergence speed varies for different CVs at different images along the path (measured by the scalar parameter *α*), it typically takes 20~40 path-optimization cycles before the CVs settle down in regions less than 0.2 Å from the corresponding converged values (Figure 5). Based on the RMSDs of MFEP, which are defined as deviations of CVs in Figure 5, averaged over all images in a given optimization cycle, we found that the path convergence is satisfactorily established after 90 path-optimization cycles, indicated by RMSDs less than 0.01 Å. Therefore, we considered that the MFEPs are essentially converged during cycles 90–100, and obtained the final free energy profiles (and standard deviations) based on the average of the free energy profiles from the last 10 path-optimization cycles.

Although the path convergence behavior may also depend on the quality of the initial path, the combination of 20 ps sampling for mean force evaluation in each image and 100 cycles of path optimization seems to be necessary to achieve a reasonable convergence of MFEP, which highlights the importance of adequate sampling in obtaining reliable free energy results. The free energy sampling task of 50 ns under this scheme is so demanding that any attempt to use ab initio QM/MM potential energy surfaces would be impractical and can only be done for much shorter timescales (see Section 4.2 for further discussion). On the other hand, free energy simulations of semiempirical QM/MM potentials are quite affordable, while maintaining this level of sampling requirement.

### 3.5. S607 Displays “Finger Motion” during ATP Hydrolysis

In our previous QM/MM calculations using reaction coordinate driving to explore potential energy surfaces, we did not detect any significant conformational change in the C-loop S607, for which the crystallographic orientation is kept throughout the potential energy scan of ATP hydrolysis (i.e., the sidechain -OH group of S607 is always H-bonded to the γ-phosphate). The dynamical information of the C-loop, as well its coupling to the chemical reaction coordinate, is therefore missing in those potential energy calculations due to the lack of entropic contributions.

Free energy sampling along the MFEPs makes it possible to examine the impact of protein dynamics on catalysis in HlyB. By analyzing the structures sampled along the MFEPs under the GAC mechanism, we identified three major conformational populations of the C-loop S607 during ATP hydrolysis, which can be categorized by the dihedral angle *d*(C-C_α_-C_β_-O_γ_) of −52°, +82°, and −172° in the Ser residue (see Supplementary Materials SM.2 for *Conformational Analysis of S607 along MFEP for ATP Hydrolysis*). The H-bonding patterns of S607 and snapshots of the representative active-site geometries for the three conformations are shown in Figure 6. Our data suggest that S607 tends to adopt distinct conformations at different stages of ATP hydrolysis.

The −52° conformation of S607, also observed in the crystal structures of the pre-hydrolysis ATP-bound HlyB [3,6], is mainly found in the reactant region of ATP hydrolysis, where S607 seems to stabilize the reactant complex RC by forming an H-bond between its sidechain -OH group and one of the non-bridging γ-phosphate oxygens in ATP (Figure 6a). The +82° conformation, in which the Ser-OH group is shared between the β- and γ-phosphates (Figure 6b), becomes populated when the system evolves toward the transition state of P_γ_-O_w_ bond formation. From an electrostatic standpoint, this local conformational change of S607 may offer an especially effective catalytic strategy for stabilizing the associative phosphoryl transition state found in HlyB, where negative charge is expected to shift to the non-bridging γ-phosphate oxygens [22,23]; such a charge buildup can be stabilized through H-bond interactions between S607-OH and the P_γ_O_3_ moiety. A flexible S607 can be useful to dynamically maintain these H-bond interactions during phosphoryl transfer. The −172° conformation is found to be dominant in the later stages of ATP hydrolysis, when the P_γ_-O_3β_ bond is cleaved with ADP and Pi is being formed. As the ADP/Pi complex formation is accompanied with the separation of the γ-phosphoryl group from ADP, a mobile S607 capable of changing its local conformation would make it possible to form alternative hydrogen bonds with the nearby acceptor groups such as β-phosphate, to compensate for the weakened H-bonding interactions with the γ-phosphate oxygens during product formation. After Pi eventually dissociates from the ADP fragment, the S607-OH is expected to stabilize ADP by shifting its H-bonds completely from γ-to β-phosphate oxygens (Figure 6c). As the C-loop Ser under this hypothesis changes its local conformation in a way similar to that of an “arginine finger” seen in many GTP/ATP hydrolyzing enzymes [15,16], we suggest that it acts as a “serine finger” for linking specific protein motion to efficient catalysis in HlyB (perhaps also in other members of ABC transporters).

### 3.6. Free Energy Profile for S607G *in Silico* Mutation

Because the C-loop Ser is not included explicitly in the collective variables we used to describe ATP hydrolysis, the response in S607 orientation suggests the existence of coupling between the protein conformational dynamics and the chemical reaction coordinate. Direct examination of the dynamical action of S607 to enzyme catalysis by experimental approaches such as mutagenesis, however, can be challenging. For HlyB, mutation of the C-loop Ser tends to destabilize the dimer interface, leading to dimer dissociation, which complicates the kinetic interpretation of the mutational effects on the chemical steps (Schmitt, personal communication). By contrast, the net effects of mutation on catalysis can be investigated computationally with less ambiguity. For example, large quaternary rearrangement of the dimer is prevented under the stochastic boundary condition used in the present simulations.

To shed light on the role of the conserved Ser residue in the LSGGQ motif, we performed additional QM/MM-MFEP simulations for the S607G mutant of HlyB-NBDs using the same computational protocols for simulating the wild-type enzyme. Because our wild-type simulations suggest that proton relay through H662 is preferred, our mutation studies are focused on the GAC mechanism. The free energy profiles for ATP hydrolysis in the wild-type HlyB and its S607G mutant are compared in Figure 3a. The comparison shows that although the free energy barrier associated with TS2 (proton transfer) is affected to a great extent, both transition states involved in phosphoryl transfer, i.e., TS3 (P_γ_-O_w_ formation) and TS4 (P_γ_-O_3β_ cleavage), are also significantly elevated in the simulated S607G mutant. This result suggests that the C-loop S607 residue stabilizes the phosphoryl-transfer transition states (likely through H-bond interactions with phosphate oxygens), whereas a mutant lacking such H-bonding capability would lead to disrupted catalysis.

## 4. Unresolved Issues

Although our QM/MM free energy simulations of HlyB-catalyzed ATP hydrolysis provide valuable insights into the enzyme mechanism in members of the ABC transporter family, our study is far from being conclusive due to the intrinsic complexity of these systems, which also poses a number of challenges to the computational models and methods we used. Some of the unresolved issues are discussed below.

### 4.1. Protonation State of H-Loop His

In the GAC mechanism, we used a neutral H662 which is singly protonated at the N_ε_ position. The recruitment of neutral His as a general acid in enzyme catalysis is not uncommon, which has been demonstrated experimentally by Lodi and Knowles for triosephosphate isomerase (TIM) [58]. For HlyB, the choice of a neutral H662 is also made based on the observation that there is no obvious H-bond acceptor near its N_δ_ position in the crystal structures. From the mechanistic point of view, a doubly-protonated His would serve as a better general acid due to a lower pK_a_ value and therefore would facilitate proton transfer to the γ-phosphate. On the other hand, because H662 is used as a catalyst in the GAC mechanism, the remaining steps to complete the catalytic cycle must also be considered. In particular, for the subsequent proton abstraction from the lytic water, the anionic species resulting from a singly protonated H662 would be a better general base than a neutral His derived from a doubly-protonated His. Therefore, given that H662 has to restore its original protonation state at the end of the catalytic cycle, there is no reason a priori for which protonation state is more advantageous from a thermodynamic perspective. A similar argument has been used by Florian and Warshel [59] in a seminal work to clarify the hydrolysis mechanism of phosphate monoester, where they showed that using an OH^−^ from pre-dissociated water for nucleophilic attack generates kinetics comparable to using water itself as a nucleophile, even though the water deprotonation step is considered thermodynamically unfavorable in terms of pK_a_.

From a simple electrostatic standpoint, doubly protonated His may help stabilize the transition state of phosphoryl transfer through interactions between the positive charge on His and the negative charge buildup on γ-phosphate oxygens [60], which could be especially effective for associative phosphoryl transfer [22]. However, the possible involvement of H662 in coupled proton transfer and phosphoryl transfer makes interpretation of H662′s role more complex, perhaps beyond a simple picture that only invokes electrostatic stabilization. To reduce the mechanistic complexity, we tested the electrostatic stabilization proposal based on the SAC mechanism with a different H662 protonation state. Comparison of the related free energy profiles in Supplementary Figure S7 shows that the overall free energy barrier actually increases from 25.0 kcal/mol when using a neutral H662 (singly-protonated at N_ε_) to 31.6 kcal/mol when using a positively-charged H662 (double-protonated at both N_ε_ and N_δ_). These results suggest that the doubly-protonated H-loop His is counter catalysis, at least for the SAC mechanism when H662 is not directly involved in proton transfer (see Supplementary Materials SM.3 for *Free Energy Results using Doubly-Protonated H662*).

### 4.2. Limitation of Methods

Free energy simulation of biological ATP hydrolysis presents a great challenge to computational enzymology. The challenge comes in part from the demand for a highly accurate PES method while maintaining its affordability, so that sufficient free energy sampling of the reactive system can be conducted [61]. Unfortunately, when ab initio (AI) QM (including density function theory, or DFT) methods are used, free energy sampling can only be performed for a very limited timescale (ps to tens of ps) due to the daunting cost of AI PES calculations.

Recently, Huang and Liao [26] computed the free energy profiles of ATP hydrolysis in the maltose transporter using the B3LYP/MM method. To avoid the time-consuming free energy sampling on the DFT/MM PES for the whole system, only dynamics of the MM particles are sampled based on the reaction path of QM atoms. A potential drawback of this approach is that the entropic contributions from the QM atoms, including the substrates and key residues in the active sites, are not consistently included in the resulting free energy profiles. The sensitivity of free energy sampling on the results can also be seen from another study by Hsu et al. [27], who reported the free energy profiles for ATP hydrolysis in the same maltose transporter based on a similar DFT/MM potential. To alleviate the computational demand for extensive equilibrium sampling, they conducted non-equilibrium free energy simulations using metadynamics (~65 ps in total duration) [27]. Although both these studies seem to suggest a dissociative phosphoryl transfer mechanism with proton transfers involving the Walker-B Glu and H-loop His residues, the free energy profiles they obtained display very different quantitative features. Huang and Liao’s simulation gives a free energy of activation of 19.2 kcal/mol [26], in good agreement with experiments, whereas Hsu et al. reported a much lower free energy barrier of 10.5 kcal/mol [27]. The large difference between the results from the two free energy simulations, in which similar DFT/MM potentials were employed to study essentially the same system, exemplifies the challenge in the reliable determination of free energy profiles for enzyme reactions.

For ABC transporter-catalyzed ATP hydrolysis, the reaction coordinate can be fairly complex, with possible involvements of protein-mediated proton relay, phosphoryl transfer, and motions of protein side chains. To avoid the demanding computation of mapping the entire multidimensional free energy surface, we obtained free energy profiles by only sampling along a one-dimensional string MFEP in CVs. To afford a total of 50 ns sampling essential for converging each MFEP (as discussed in Section 3.4), we focused on the QM/MM potential using the semiempirical (SE) AM1 molecular orbital method, which has been used widely in enzyme simulations [62,63].

A major limitation of the AM1 method, however, is the lack of an explicit description of *d*-orbital, which is important for modelling phosphoryl transfer reactions. Although our simulations suggest an associative pentacovalent phosphoryl transfer intermediate (IM3 in GAC; ~−6.0 kcal/mol), this prediction should be taken with caution as AM1 tends to overstabilize phosphorane geometry [64,65]. Despite the fact that special SE methods such as AM1/d-PhoT [65] have made enzyme simulations with explicit *d*-orbital treatment for phosphorous possible [41,66], PES methods with AI QM-quality but maintaining the efficiency of SE methods would be highly desirable. These may be achieved by multiscale approaches, such as reaction path force matching (RP-FM) [61], density-functional tight-binding based force matching [67], multiple time step integration [68], and machine learning [69], in combination with advanced enhanced sampling techniques [70,71]. Another limitation of the present work is the use of a stochastic boundary condition to reduce the computational cost; therefore, long-range electrostatics is lacking. Future investigations are needed to verify the GAC mechanism using periodic boundary condition simulations with explicit long-range QM/MM electrostatic treatments [67,72,73,74].

### 4.3. Solvent Kinetic Isotope Effects

Solvent kinetic isotope effects (KIE) have been reported for HlyB-NBD with a *k*_cat,H2O_/*k*_cat,D2O_ value of 0.79 [3], and for the Rad50 ABC protein in the range of 0.92–0.98 [75]. The inverse KIE found in HlyB or generally the lack of KIE in these ABC systems implies that the proton abstraction from water is not rate limiting. Our earlier potential energy profiles [14] could not explain the inverse KIE because the rate-limiting step (TS3 in GAC) involves transferring a proton from the lytic water, making the overall rate isotopically dependent on the solvent. With the entropic contributions included in the present work, the rate-determining free energy barrier becomes the proton transfer step from H662 to ATP (TS2 in GAC), whereas the proton transfer from water has a lower free energy barrier; therefore, the free energy profile computed for the GAC mechanism is now compatible with the observed solvent KIE results. It is noted that nuclear quantum effects are still missing in our simulations. To elucidate the origin of the solvent KIE, an explicit description of quantized vibration and multidimensional tunneling [76] will be needed, which may impact the multi-step enzyme mechanism in HlyB in a more complicated manner, and therefore we leave the subject for future investigation.

## 5. Concluding Remarks

In this article, making use of the QM/MM MFEP simulations, we have confirmed our earlier conclusion based on QM/MM potential energy calculations that the H-loop residue H662 in HlyB-NBDs catalyzes ATP hydrolysis by providing a “chemical linchpin” for assisted proton transfer. The GAC reaction mechanism is in accord with the pH dependence of the ATPase activity reported for HlyB, which shows maximal ATPase activities at pH = 7 [3], a condition necessary for His to exist predominantly as a singly protonated neutral species to activate the proton relay pathway.

The H-loop mediated GAC mechanism can be used to rationalize the loss of ATPase activity seen in mutants of HlyB (H662A, <0.1% residual ATPase activity) [3,5] and other ABC transporters, such as the histidine permease HisP (H211D, H211R, and H211Y, <2% residual ATPase activity) [10,77] and the maltose transporter MalK (H192R, <2% residual ATPase activity) [11]. Such substitutions can be detrimental to the GAC proton relay pathway for the lack of an exchangeable proton in the suitable pKa range. Consistently, spectroscopic studies based on electron paramagnetic resonance (EPR) and biochemical experiments show that the H-loop mutant H573A of the lipid transporter MsbA reduces the ATPase activity to 8% of the wild-type enzyme [12].

By contrast, in the multidrug exporter Pdr5, H-loop His seems to be non-essential for hydrolysis, with the H1608A mutant retaining ATPase activity but selectively disabling the transport of rhodamine in the intact transporter [13]. If assisted proton transfer is a conserved feature for ABC-mediated ATP hydrolysis, as suggested from our simulations, there might be alternative proton relay mechanisms in the absence of H-loop His. We noticed that solvent-assisted proton transfers have been discussed for other ATP hydrolyzing enzymes, such as F_1_-ATPase [78], PcrA helicase [79], DNA polymerase [80], myosin [81], and actin [82]. Therefore, one possible rescuing mechanism in the mutated systems to compensate for the loss of His-mediated proton relay is to recruit a solvent water molecule to the active-site pocket originally occupied by the His side chain; such a bridging water may be used to relay a proton to avoid the unfavorable direct proton transfer pathway in SAC.

Recent luminescence resonance energy transfer studies of the bacterial ABC system MJ0796 by Zoghbi and Altenberg [83] suggest that hydrolysis at only one ATP-binding site is sufficient to drive its homodimeric NBDs to dissociate. Intriguingly, heterodimeric NBDs are found in many asymmetric ABC transporters (including a number of eukaryotic ABC exporters), in which mutations on residues essential to ATP hydrolysis occur naturally, making the two active sites functionally non-equivalent [13,60,84,85,86,87,88]. For example, in human transporters associated with antigen processing (TAP), the H-loop in the TAP1 NBD subunit contains an H/Q mutation compared with the canonical H-loop in the TAP2 subunit [60]. The H/Q mutation in TAP1, combined with an E/D mutation in Walker B of the same subunit and an LSGGQ → LAA/VGQ mutation in the C-loop of TAP2, renders a “degenerate” ATP-binding site devoid of ATPase activity [60,89]. The present work provides insights into the mechanism by which ATP hydrolysis is disabled in the degenerate site: the H/Q mutation may contribute to the diminished ATP hydrolysis activity by shutting down the essential proton relay pathway; mutations on the C-loop may decouple the “serine finger”, which provides electrostatic stabilization dynamically, from the chemical steps involving phosphoryl transfer.

## Figures and Tables

**Table 1:** Free energies (in kcal/mol) and collective variables (in Å) of the stationary states on the MFEP obtained for the GAC mechanism.

State	*α*	Δ*G*	P_γ_-O_3β_	P_γ_-O_W_	H_N_-N_ε,H662_	H_N_-O_2γ_	H_W_-O_W_	H_W_-N_ε,H662_
RC	0.00	0.0	1.67	3.92	1.00	2.07	0.97	3.52
TS1	0.15	9.6	1.68	2.79	1.00	2.10	0.97	3.09
IM1	0.23	2.9	1.73	2.15	1.01	1.89	0.97	3.01
TS2	0.31	14.0	1.69	2.00	1.27	1.31	0.97	2.91
IM2	0.43	2.9	1.68	1.95	2.19	0.96	0.97	2.75
TS3	0.68	12.0	1.80	1.74	3.43	0.95	1.26	1.31
IM3	0.77	−6.0	1.93	1.67	3.45	0.95	1.98	1.01
TS4	0.89	5.5	2.89	1.62	3.65	0.96	2.20	1.00
PC	1.00	−1.7	3.80	1.60	3.45	0.95	2.22	1.00

**Table 2:** Free energies (in kcal/mol) and collective variables (in Å) of the stationary states on the MFEP obtained for the SAC mechanism.

State	*α*	Δ*G*	P_γ_-O_3β_	P_γ_-O_W_	H_W_-O_W_	H_W_-O_2γ_
RC	0.00	0.0	1.66	3.88	0.96	3.44
TS1	0.14	9.1	1.69	2.97	0.97	3.10
IM1	0.31	1.2	1.71	2.01	0.97	2.68
TS2	0.50	25.0	1.71	1.90	1.18	1.35
IM2	0.66	−14.9	1.85	1.68	2.22	0.95
TS3	0.73	−14.1	1.83	1.67	2.70	0.95
IM3	0.76	−14.2	1.90	1.68	2.88	0.95
TS4	0.91	−4.3	2.88	1.62	3.27	1.00
PC	1.00	−12.2	3.57	1.60	3.31	1.02

## Supplementary Materials

The following are available online. Committor analysis, conformational analysis of S607 along MFEP for ATP hydrolysis, and free energy results using doubly-protonated H662 are available.
