# Supplementary material for: Mapping Free Energy Pathways for ATP Hydrolysis in the E. coli ABC Transporter HlyB by the String Method
Source: Molecules. 2018 Oct 16;23(10):2652. doi: 10.3390/molecules23102652 (PMC6222333; doi:10.3390/molecules23102652)
Supplement: Supplementary file 1 [file molecules-23-02652-s001.pdf]

October 15, 2018

Supplementary Materials

Mapping Free Energy Pathways for ATP Hydrolysis in the *E. coli* ABC Transporter HlyB by the String Method

*Yan Zhou, Pedro Ojeda-May, Mulpuri Nagaraju, Bryant Kim, and Jingzhi Pu**

Department of Chemistry and Chemical Biology, Indiana University-Purdue University Indianapolis, 402 N. Blackford St., Indianapolis, IN 46202

* To whom correspondence should be addressed. Email: jpu@iupui.edu

1. Committor analysis for transition states located on the MFEPs

To verify the locations of the transition state ensembles along the string paths, we conducted committor probability analysis based on a large number of short trajectories launched at the transition state configurations.

Starting from the MFEP obtained after 100 cycles of string optimization, QM/MM MD simulations of 50 ps were first carried out to prepare ensembles of initial configurations for subsequent computation of committor distributions at TS2 (proton transfer from H662 to ATP) and TS3 (concerted proton transfer from water to H662 and Pγ-O_W formation) for the GAC and TS2 (concerted proton transfer from water to ATP and Pγ-O_W formation) for the SAC mechanism. During these equilibrium simulations, the six (or four) collective variables in the GAC (or SAC) mechanism were restrained to the corresponding values at the transition states. The force constant used in the harmonic restraints is 2,000 kcal/mol/Å^2. As a result, 500 configurations were saved in each simulation. For each of the 500 configurations sampled for the transition-state ensemble, we performed 100 independent simulations of 0.1 ps in length, initiated with different random seeds and without any restraints on the collective variables. This leads to a collection of 50,000 short trajectories launched at each transition state, based on which the probabilities for the trajectories to go forward to the product side and backward to the reactant side were computed. The histograms of configurations that give similar probabilities were obtained and plotted in Figures S1-S3 with bin sizes of 0.1 and 0.05. For comparison, the numerical results were also fit to Gaussian distributions.

Let $p_i \in [0,1]$ represent the probability of the trajectory that commits to the product, and n_i represents the number of occurrence at a certain probability, where $i \in [1,N]$ and N is the total number of bins. n_i follows the relationship, $\sum_{i=1}^{N} n_i = 500$. The average probability is calculated by $\bar{p} = \sum_{i=1}^{N} n_i p_i / 500$. The standard deviation of the probability is calculated by

$\sigma = \sqrt{\sum_{i=1}^{N} n_i (p_i - \bar{p})^2 / 499}$. The unnormalized Gaussian distribution is calculated by $f_i(p_i) = e^{-(p_i - \bar{p})^2/2\sigma^2} \Big/ (\sigma\sqrt{2\pi})$. The distributions based on the Gaussian distribution are estimated by $n_i^{\mathrm{G}} = 500 f_i \Big/ \sum_{j=1}^{N} f_j$. An ideal transition state ensemble is expected to generate a distribution that peaks at 0.5.

Figure S1 shows the committor probability distributions at TS2 located on the GAC MFEP. The probability distribution peaks at 0.46 and 0.47, for the bin size of 0.1 and 0.05, respectively. Figure S2 displays the committor probability distribution at TS3 on the GAC MFEP. The peaks of the distribution both occur at 0.51 for the two bin sizes tested. Figure S3 shows the committor probability distributions at TS2 located on the SAC MFEP, which peaks at a value of 0.45 and 0.46, for the bin size of 0.1 and 0.05, respectively. The numerical values of the average committor probabilities (approximately corresponding to the peak distribution) and standard deviation of the distribution for each case are given in Tables S1-S3. These committor probability distributions indicate that our MFEP calculations indeed locate the transition states correctly.

Table S1. The peak committer probability and standard deviation computed for TS2 located on the GAC MFEP (with bin sizes of 0.1 and 0.05).

Bin size	$\bar{p}$	σ
0.1	0.46	0.22
0.05	0.47	0.22

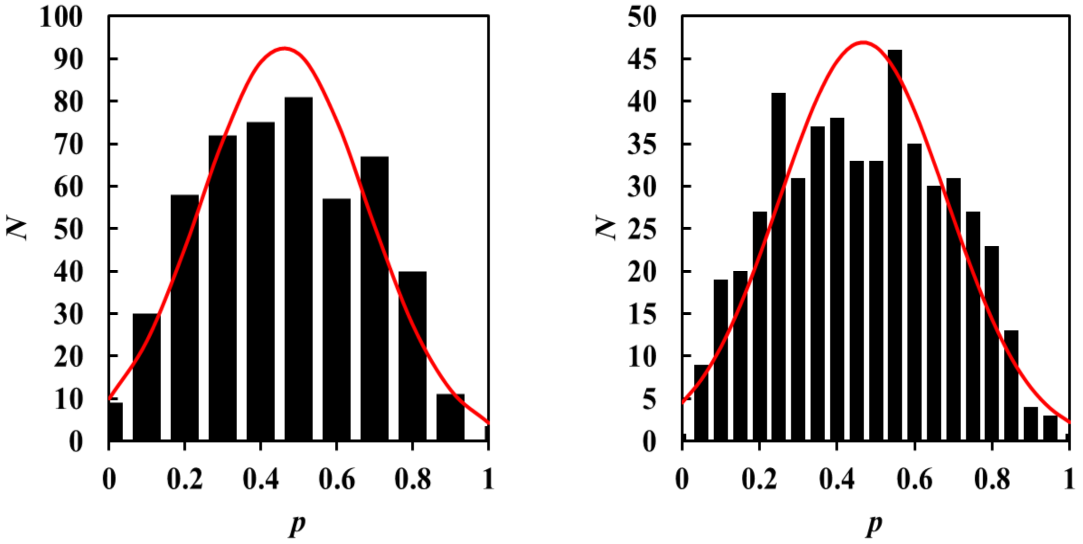

Figure S1. Committor probability distributions at TS2 located on the GAC MFEP with bin sizes of 0.1 (left) and 0.05 (right). Gaussian fit of each distribution is shown in red.

Table S2. The peak committer probability and standard deviation computed for TS3 located on the GAC MFEP (with bin sizes of 0.1 and 0.05).

Bin size	$\bar{p}$	σ
0.1	0.51	0.24
0.05	0.51	0.24

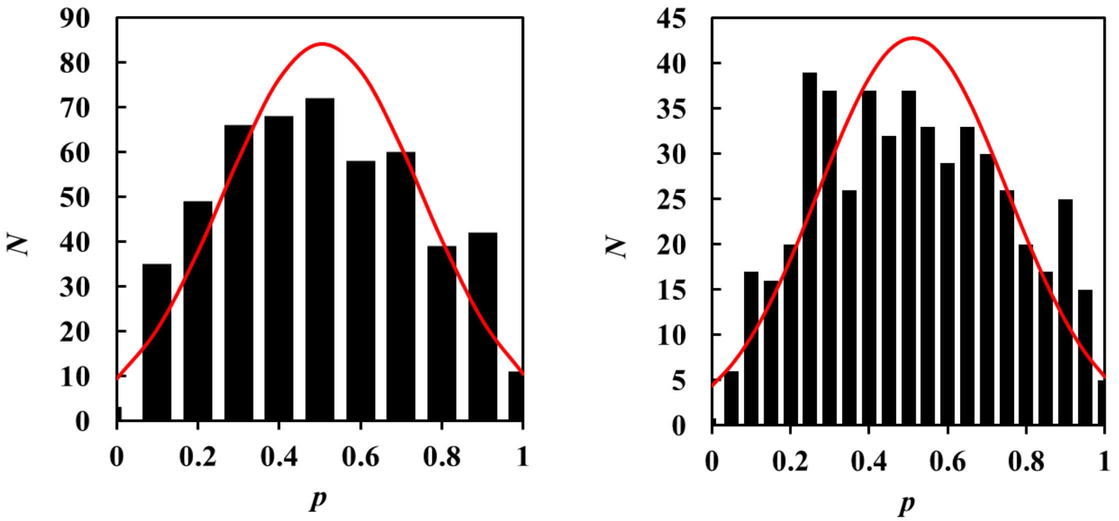

Figure S2. Committor probability distributions at TS3 located on the GAC MFEP with bin sizes of 0.1 (left) and 0.05 (right). Gaussian fit of each distribution is shown in red.

Table S3. The peak committer probability and standard deviation computed for TS2 located on the SAC MFEP (with bin sizes of 0.1 and 0.05).

Bin size	$\bar{p}$	σ
0.1	0.45	0.24
0.05	0.46	0.24

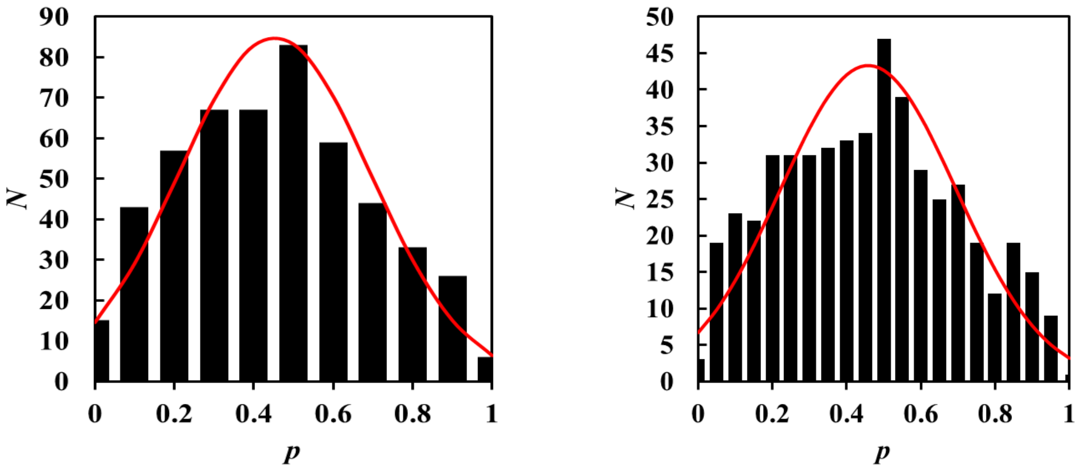

Figure S3. Committor probability distributions at TS2 located on the SAC MFEP with bin sizes of 0.1 (left) and 0.05 (right). Gaussian fit of each distribution is shown in red.

2. Conformational analysis of S607 along MFEP for ATP hydrolysis

To analyze the local conformation of the C-loop S607 in ATP hydrolysis, we obtained the histograms of the normalized distribution of the dihedral angle d(C-C$_\alpha$-C$_\beta$-O$_\gamma$) sampled along the MFEP under the GAC mechanism, which reveal three major conformational populations at -52˚, +82˚, and -172˚ (Figure S4). The H-bonding patterns and representative geometries for these three distinct conformations can be found in Figure 6 in the main text.

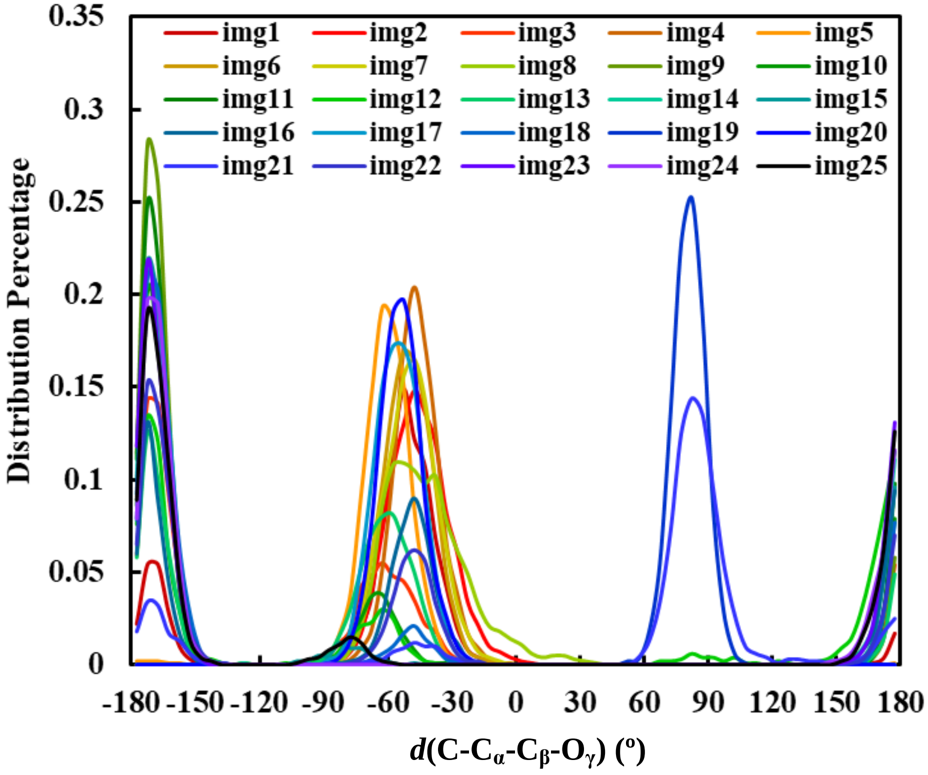

Figure S4. The normalized distributions of the dihedral angle d(C-C$_\alpha$-C$_\beta$-O$_\gamma$) of the C-loop residue S607 in chain A of HlyB-NBDs sampled along the minimum free energy path (MFEP) under the GAC mechanism.

3. Free energy results using doubly-protonated H662

To test whether the different protonation state of the H-loop His generates any significant impact on the free energy profile of ATP hydrolysis, we also conducted free energy simulations for the SAC mechanism (due to its simplicity) by using a doubly protonated H662. To simulate the doubly-protonated His, the positively charged CHARMM residue HSP is used to replace the neutral His residue HSE (singly protonated at the N$_\varepsilon$ position) when setting up the HlyB system. For the HSP system, we follow the same simulation protocols described in the main text for obtaining MFEPs for the HSE system: 20 ps QM/MM MD sampling was carried out for each image during each path-optimization cycle, and 100 cycles of path optimization were performed. The free energy profile and progressions of the distance-based collective variables for the HSP system

under the SAC mechanism are shown in Figures S5 and S6. The free energy profiles for the HSP and HSE systems under the SAC mechanism are compared in Figure S7 and Table S4. The free energy of activation for the rate-limiting step at TS2 is 31.6 and 25.0 kcal/mol for the HSP and HSE systems, respectively, suggesting that using the doubly-protonated H662 is counter catalysis compared with the neutral H662.

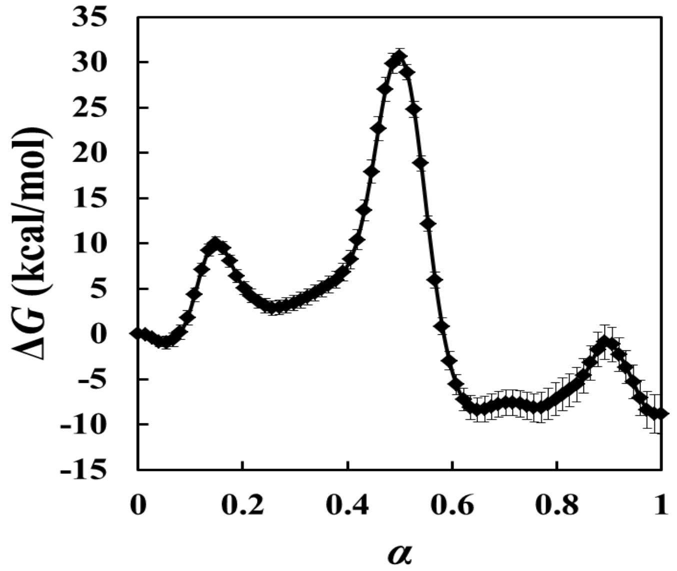

Figure S5. The free energy profile for the SAC mechanism in chain B of HlyB-NBDs with doubly-protonated H662 (HSP).

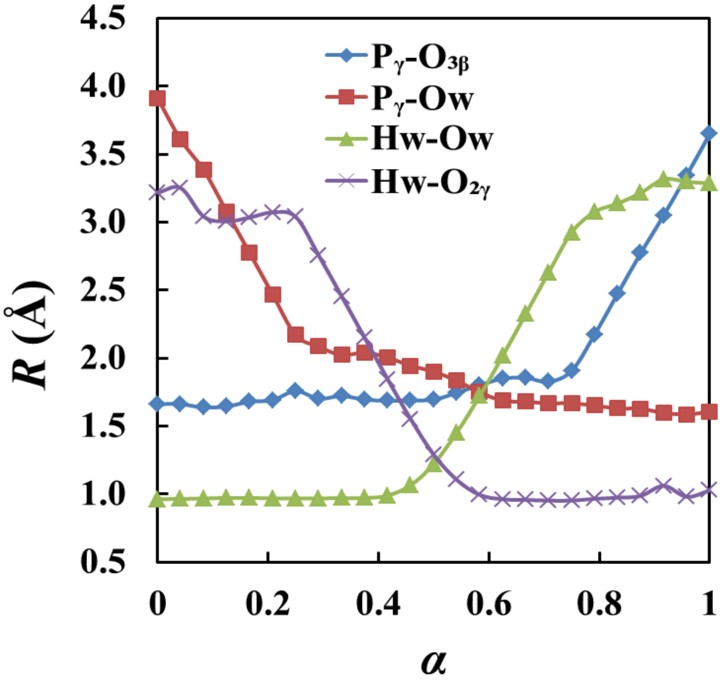

Figure S6. Progressions of the four distance-based collective variables along the MFEP under the SAC mechanism using the doubly-protonated H662 (HSP).

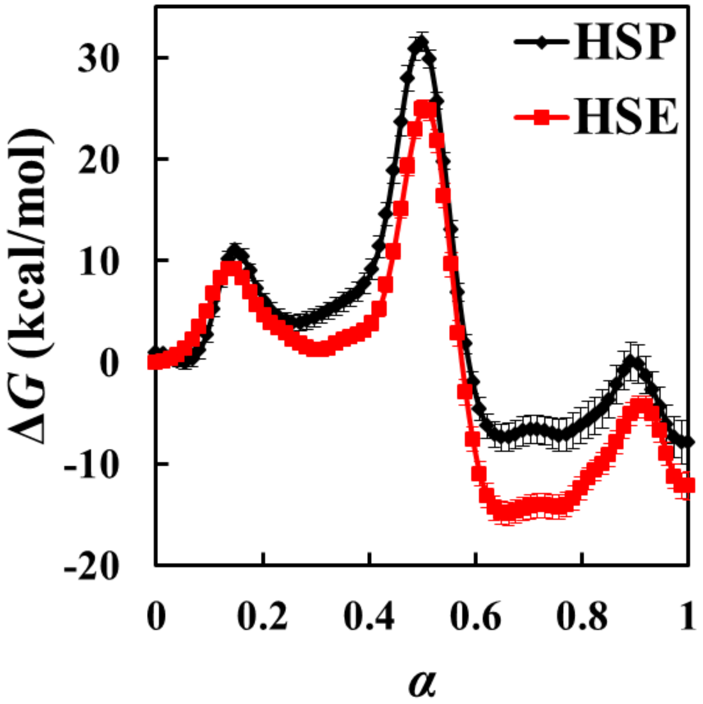

Figure S7. Comparison of the free energy profiles under the SAC mechanism in chain B of HlyB-NBDs with H662 being doubly protonated (HSP) and singly protonated at the N_ε position (HSE), respectively.

Table S4. Comparison of free energies of the stationary states located on the SAC MFEPs in chain B of HlyB-NBDs with H662 being HSP and HSE, respectively.

	RC	TS1	IM1	TS2	IM2	TS3	IM3	TS4	IM5
HSP	0.0	11.0	3.8	31.6	-7.4	-6.6	-7.2	0.1	-7.9
HSE	0.0	9.1	1.2	25.0	-14.9	-14.1	-14.2	-4.3	-12.2
